# Thanks to all those who reviewed for *European Journal of Medical Research* in 2015

**DOI:** 10.1186/s40001-016-0200-6

**Published:** 2016-02-15

**Authors:** Dieter Häussinger, Johannes Waltenberger

**Affiliations:** Clinic for Gastroenterology, Hepatology and Infectious Diseases, Heinrich Heine University, 40225 Düsseldorf, Germany; Department of Cardiology, Cardiovascular Research Institute Maastricht (CARIM), Maastricht, The Netherlands; Department of Cardiovascular Medicine, University of Muenster, Muenster, Germany; Cells-in-Motion Cluster of Excellence (EXC 1003–CiM), University of Münster, Münster, Germany

A peer-reviewed journal would not survive without the generous time and insightful comments of the reviewers, whose efforts often go unrecognized. Although final decisions are always editorial, they are greatly facilitated by the deeper technical knowledge, scientific insights, understanding of social consequences, and passion that reviewers bring to our deliberations. For these reasons, the Editors-in-Chief and staff of *European Journal of Medical Research* warmly thank the 243 reviewers whose comments helped to shape the journal, for their invaluable assistance with review of manuscripts in Volume 20 (2015).

**Mohammed Al-Sofiani**

USA

**Hagen Andruszkow**

Germany

**Stefano Angioni**

Italy

**Joan Albert Arnaiz**

Spain

**Michael Ayad**

USA

**Yener Aydin**

Turkey

**Erman Aytac**

Turkey

**Lindsey Baden**

USA

**Ingo Banke**

Germany

**Kelli Barbour**

USA

**Antonio Bellastella**

Italy

**Arne Berner**

Germany

**Peter Biberthaler**

Germany

**Matthias Biebl**

Germany

**Elena Bignami**

Italy

**Ugur Bilge**

Turkey

**Sabine Bischoff**

Germany

**Mark Borsody**

USA

**David Borsook**

USA

**Emanuela Bostjancic**

Slovenia

**Gerardo Botti**

Italy

**Stephan Brand**

Germany

**Norman Briffa**

UK

**Olga Bushueva**

Russian Federation

**Jonas Bystrom**

UK

**Lu Cai**

USA

**Yun-Xia Cao**

China

**Laura Capoccia**

Italy

**Massimo Caputo**

UK

**Rhanderson Cardoso**

USA

**Abel Casso Dominguez**

USA

**Antonio Celada**

Spain

**Yi-Chun Chen**

Taiwan

**Yoshihiko Chiba**

Japan

**Norio Chihara**

USA

**Shubhada V Chiplunkar**

India

**Ya-Wen Chiu**

Taiwan

**George Chrousos**

Greece

**Sally Collins**

UK

**Fernanda Marciano Colombo**

Brazil

**Eric Corty**

USA

**M Czarnowska-Cubała**

Poland

**Surabhi Dangi-Garimella**

USA

**Mgj De Boer**

The Netherlands

**Patrick Delhey**

Germany

**Jorge Dias**

Portugal

**Max Dieterich**

Germany

**Amolkumar Diwan**

India

**Dimitrios Dragoumis**

UK

**Shkelzen Duci**

Albania

**Georges El Hayek**

USA

**Timothy England**

UK

**Hasan Erdogan**

Turkey

**Fatih Ermis**

Turkey

**Özgen Eser**

Turkey

**Gamal Esmat**

Egypt

**Emiliano Fabiani**

Italy

**Claudius Falch**

Germany

**Micha Feld**

Germany

**Serhiy Forostyak**

Czech Republic

**Michael Frink**

Germany

**Michael Froehner**

Germany

**Maria Angeles Gálvez**

Spain

**Li Gaofeng**

China

**Junbo Ge**

China

**Andrea Gioffredi**

Italy

**Raphael Giraud**

Switzerland

**Antje Gottschalk**

Germany

**Ever Grech**

UK

**J Guarne**

USA

**Carl Haasper**

Germany

**Johannes Haberle**

Switzerland

**Daniel Habermehl**

Germany

**Gabriel Halat**

Austria

**Alexander Tobias Haug**

Germany

**Hakon Haugaa**

Norway

**Ward Heggermont**

Belgium

**Christian Herren**

Germany

**Julie Hicks**

USA

**Yukihito Higashi**

Japan

**Frank Hildebrand**

Germany

**Tzong-Shiann Ho**

Taiwan

**Norman Hoff**

Germany

**Martijn Hofman**

The Netherlands

**Christian Hollinsky**

Austria

**Klemens Horst**

Germany

**Aihua Hu**

USA

**Weijian Huang**

China

**Markus Huber-Lang**

Germany

**Markos Ioannou**

Greece

**Kannamannadiar Jayaprakasan**

UK

**Ze-Xu Jiao**

USA

**Ekaterina S Jordanova**

The Netherlands

**Thomas Kammer**

Germany

**Shan Kang**

China

**Stefanie Keymel**

Germany

**Bernhard Klumpp**

Germany

**Matthias Knobe**

Germany

**Takashi Kohno**

Japan

**Igor Koncar**

Serbia

**Shin-Ichi Kosugi**

Japan

**Mohamed Kotb El Sayed**

Egypt

**Tobias M Kraus**

Germany

**Shashi Kumar**

USA

**Jolanta Kupryjanczyk**

Poland

**Wei Lai**

China

**Christian Lanckohr**

Germany

**Giovanni Landoni**

Italy

**Risha Lane**

UK

**Philipp Lechler**

Germany

**Hao Li**

China

**Jun Li**

Germany

**Q Q Li**

China

**Philipp Lichte**

Germany

**Miriam Lichtner**

Italy

**Juergen Liebermann**

USA

**Lucille London**

USA

**John Lurain**

USA

**Thomas Lustenberger**

Germany

**Yoshihiko Maehara**

Japan

**Am Malagoni**

Italy

**Josep Mallolas**

Spain

**Christos Maniotis**

Greece

**Robert Mankowski**

The Netherlands

**Sebastian Manncke**

Germany

**Lorella Marinucci**

Italy

**Paolo Martelletti**

Italy

**Maria Paola Martelli**

Italy

**Mar Masia**

Spain

**Vikram Mathews**

India

**Christiane Matuschek**

Germany

**Franz Mayer**

Austria

**Marc Merx**

Germany

**Ruth Miller**

USA

**Ines Monte**

Italy

**Ho Sik Moon**

Korea, Republic of

**Hector Mora-Montes**

Mexico

**Sonia Moretti**

Italy

**Christian Mueller**

Germany

**Silvio Nadalin**

Germany

**Kazuhiko Nakabayashi**

Japan

**Ravi Prasad Avati Nanjundappa**

USA

**Lukas Negrin**

Austria

**Thomas Neuhaus**

Germany

**Claudia Neunaber**

Germany

**Antonio Nouvenne**

Italy

**Andreas Nussle**r

Germany

**Funlayo Odejinmi**

UK

**Gabor Olah**

USA

**Georg Omlor**

Germany

**Ali Oto**

Turkey

**Noriko Ouji-Sageshima**

Japan

**Yong-Han Paik**

Korea, South

**Ana Palei**

Brazil

**Lucia Palmisano**

Italy

**Utpal Pandya**

USA

**Vibhav Parghi**

India

**Saverio Parisi**

Italy

**Parham Parto**

USA

**Andrzej Pawlik**

Poland

**Stephan Payr**

Austria

**Alexander Petter-Puchner**

Austria

**Andor Pivarcsi**

Sweden

**Ruben Plentz**

Germany

**M J Post**

The Netherlands

**Peter Prodinger**

Germany

**Raquel Rabionet**

Spain

**Shawn Ragbir**

USA

**Tienush Rassaf**

Germany

**Björn Rath**

Germany

**Borna Relja**

Germany

**Johannes Rey**

Germany

**Ricardo Ribeiro**

Portugal

**Wiltrud Richter**

Germany

**Roberta Rizzo**

Italy

**Anderson Rodrigues**

Brazil

**Becerra S Patricia**

USA

**Soya Sam**

USA

**Tírcia Santos**

Portugal

**Philip Savage**

UK

**Katja Schenke-Layland**

Germany

**Martin Schick**

Germany

**Orazio Schillaci**

Italy

**Thomas Schmid**

Austria

**Werner Schmoelz**

Austria

**Karl-Werner Schweppe**

Germany

**Henrik Schytz**

Denmark

**Ludovica Segat**

Italy

**Renato Seracchioli**

Italy

**Ege Can Serefoglu**

Turkey

**Giuliano Sette**

Italy

**Mohamed Shawarby**

Saudi Arabia

**Yueping Shen**

China

**Takehiko Shimoyama**

Japan

**Sebastian Siebenlist**

Germany

**Gautam Sikka**

USA

**Martin Sillem**

Germany

**Ivone Silva**

Portugal

**Seema Singhal**

India

**Tea Skaaby**

Denmark

**John Skedros**

USA

**Pierre Smeesters**

Belgium

**George Smith**

USA

**Jose Tarfur Soto**

USA

**Linda Sousse**

USA

**Venkateshan S P**

India

**Vanessa Stadlbauer**

Austria

**Carsten Stephan**

Germany

**Tomasz Stompór**

Poland

**P N Sylaja**

India

**Zi-Hui Tang**

China

**Brandie Taylor**

USA

**Bijin Thajudeen**

USA

**Alfonso Tolentino**

USA

**Yeqing Tong**

China

**Tatsuo Torizuka**

Japan

**Natalie Torok**

USA

**Pelagia Tsoutsou**

Greece

**Ronald van Vollenhoven**

Sweden

**Katerina Vassiou**

Greece

**Helen Veste**r

Germany

**Lorenz Wanke-Jellinek**

USA

**Sugiko Watanabe**

Japan

**Christian Weber**

Germany

**Ching-Fu Weng**

Taiwan

**Matthias Weuster**

Germany

**D Wichmann**

Germany

**Maria Witte**

Germany

**Heinrich Worth**

Germany

**William Wu**

USA

**Karin Wuertz-Kozak**

Switzerland

**Lei Yao**

China

**Chun-Ting Yeh**

Taiwan

**Yasar Yildirim**

Turkey

**Leimiao Yin**

China

**Paul Zarogoulidis**

Greece

**Christian Zeckey**

Germany

**Keqin Zhang**

China

**Jin an Zhang**

China

**Xiufen Zheng**

Canada

**Linuo Zhou**

China

**Amir Ziaee**

Iran

